# Kyste hydatique du foie rompu dans la paroi abdominale et dans le muscle psoas : à propos d'une rare observation

**DOI:** 10.4314/pamj.v10i0.72207

**Published:** 2011-09-07

**Authors:** Issam En-Nafaa, Mountassir Moujahid, Abdelouahabe Alahyane, Touria Amil, Ahmed Hanine, Tarik Ziadi

**Affiliations:** 1 Service de radiologie, HMIMedV, Rabat Maroc; 2 Service de chirurgie viscérale, HMIMedV, Rabat Maroc

**Keywords:** Kyste hydatique, rupture, abdomen, foie, psoas, Maroc

## Abstract

Le kyste hydatique du foie est une parasitose qui sévit à l′état endémique au maroc. La rupture dans la paroi abdominale et dans le psoas est une complication exceptionnelle. Nous rapportons un cas de kyste hydatique du foie rompu dans la paroi et dans le muscle psoas. Le diagnostic a été établi sur les données de l′échographie et surtout de la tomodensitométrie. Le patient a été opéré avec des suites simples.

## Introduction

L'hydatidose est une anthropozoonose qui sévit à l’état endémique au Maroc. La localisation hépatique reste de loin la plus fréquente et se voit dans 70% des cas environ [1]. Sa gravité tient surtout de ses complications essentiellement l'infection et la rupture dans les voies biliaires. La rupture d'un kyste hydatique (KH) dans la paroi abdominale est une complication exceptionnelle. Nous rapportons le cas d'un KH du foie rompu dans la paroi abdominale et dans le muscle psoas.

## Observation

Patient de 43 ans, sans antécédents pathologiques notables, présentait depuis trois jours une volumineuse masse douloureuse de l'hypochondre droit qui s'est fistulisée secondairement, évoluant dans un contexte de fièvre non chiffrée. L'examen a trouvé un patient fébrile à 38,5°, présentant une masse de l'hypochondre droit étendue au flanc, fluctuante, douloureuse, fixe par rapport au plan superficiel avec rougeur et chaleur de la peau et présence d'une fistule cutanée en regard. Le reste de l'examen clinique était sans particularité. Le bilan biologique trouvait une hyperleucocytose, une augmentation de la CRP.

Une échographie abdominale a été réalisée et a montré une collection liquidienne multi loculée de la paroi abdominale antérolatérale droite communicant avec une masse hépatique périphérique sous capsulaire à paroi calcifiée par endroits et à contenu hétérogène. Avec mise en évidence d'une autre collection liquidienne d'allure abcédée intéressant le muscle psoas droit communiquant avec la collection pariétale.

**Figure 1:**
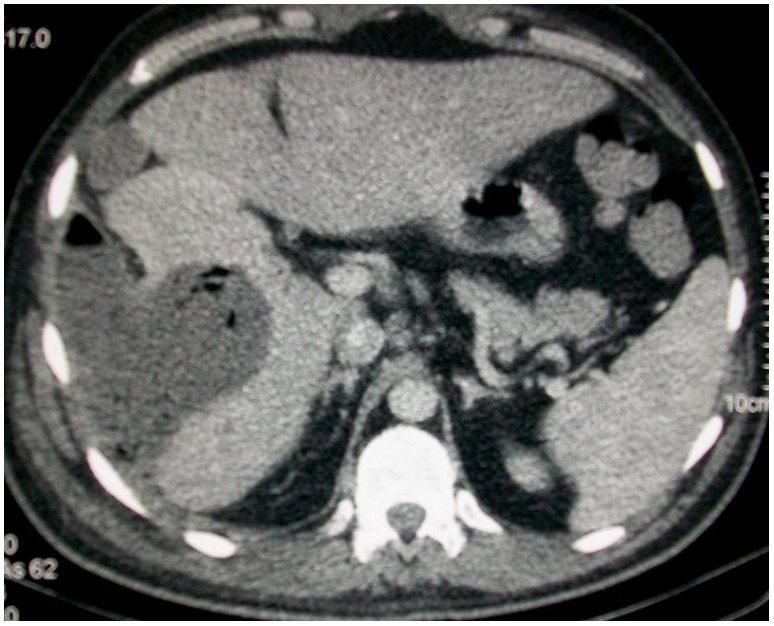
Tomodensitométrie abdominal en coupes axiales après injection de produit de contraste iodé, en fenêtre parenchymateuse montrant une lésion hypodense du foie, à paroi calcifiée par endroit et à contenu hétérogène renfermant des bulles d'air

**Figure 2:**
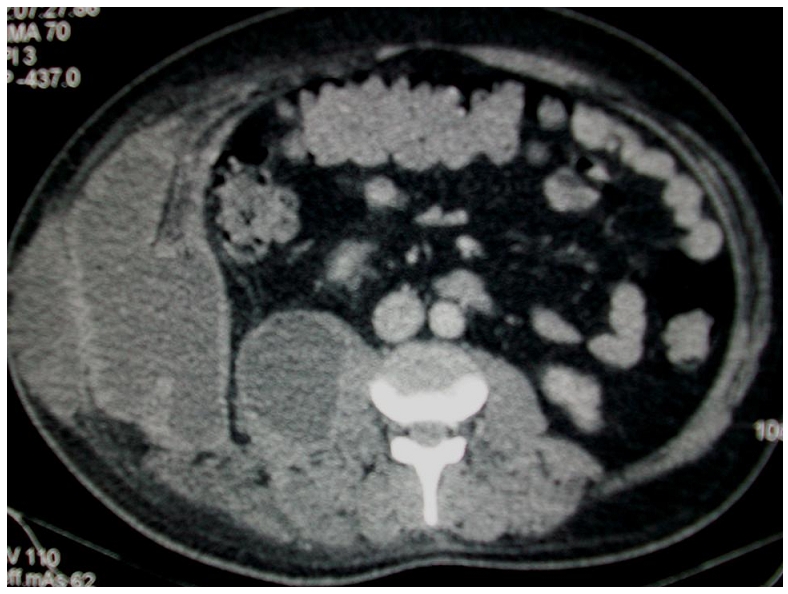
Tomodensitométrie abdominal en coupes axiales après injection de produit de contraste iodé, en fenêtre parenchymateuse montrant la présence de lésions hypodenses de la paroi abdominale et du muscle psoas droit avec une collection sous cutanée

**Figure 3:**
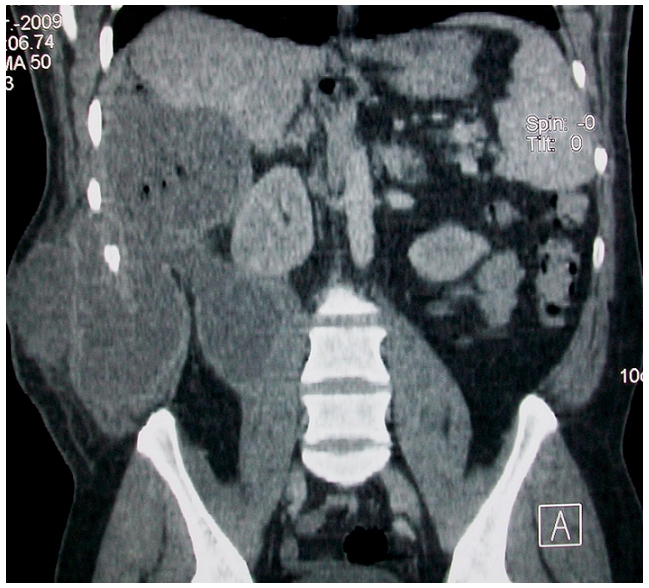
Tomodensitométrie abdominal en reconstructions sagittales après injection de produit de contraste iodé, en fenêtre parenchymateuse montrant la communication entre la lésion hépatique et les lésions pariétales, ainsi qu'avec le muscle psoas droit

Une tomodensitométrie abdominale (Figure 1, Figure 2, Figure 3) a été réalisée en acquisition spiralée avant et après injection de produit de contrast iodé avec reconstructions multi planaires et a objectivé la présence d'une lésion hypodense du foie intéressant les segments VI, VII, et VIII, bien limitée dont les parois sont calcifiées par endroit et dont le contenu est hétérogène renfermant de multiples bulles d'air. Cette lésion mesurait 70mm×60mm environ de grands axes, était en continuité avec une lésion hypodense intéressant les muscles larges de l'abdomen et le psoas droit, avec présence d'une collection liquidienne hétérogène sous cutanée en regard.

Le diagnostic d'un KH hépatique surinfecté et fistulisé dans la paroi abdominale et compliqué d'un abcès sous cutané, des muscles larges et du psoas droit a été posé. Le traitement était chirurgical avec des suites opératoires simples.

## Discussion

Le kyste hydatique (KH) est une anthropozoonose très fréquente au Maroc, elle est due au développement de la forme larvaire du tænia du chien ecchinococcus granulosis. Elle sévit sous forme endémique dans les pays du bassin méditerranéen, le moyen orient, l'Amérique du sud, l'Australie, la nouvelle Zélande et l'Afrique de l'est [1]. Le KH peut siéger dans n'importe quelle partie de l'organisme mais la localisation hépatique reste la plus fréquente (70%), suivie de l'atteinte pulmonaire (10 à 40%) [1].

L'atteinte est le plus souvent asymptomatique et la symptomatologie clinique est variable, elle dépend du siège du kyste, sa taille, son stade de développement et à la présence ou non de complications, la douleur abdominale reste le symptôme clinique le plus fréquent [1].

Les complications les plus courantes sont l'infection du contenu du kyste et la rupture dans les voies biliaires qui se voit dans 5à 10% des cas [1]. La rupture dans la paroi abdominale est une complication exceptionnelle, elle est retrouvée dans 0,1 à 1,5% des cas [2]. Moins de dix cas sont rapportés dans la littérature [3] et la rupture dans le muscle psoas n'a jamais été décrite à notre connaissance. Elle est la conséquence d'un double facteur, mécanique et inflammatoire, d'une part il y a l'infection du contenu du kyste entrainant l'inflammation du perikyste et la symphyse de ce dernier avec la paroi abdominale, d'autre part l’érosion progressive de la paroi abdominale par un perikyste épais et calcifie favorisée par les mouvements respiratoires [3]. Dans tous les cas décrits le kyste était en contact avec la paroi abdominale et la fistulisation était le mode de révélation [4]. La symptomatologie consiste en une masse abdominale pariétale douloureuse et fébrile associée ou non à un ictère cutanéo-muqueux. La biologie permet de mettre en évidence une hyperleucocytose et une élévation de la CRP témoignant de l'infection et une perturbation du bilan hépatique en cas de complication biliaire [5].

Le diagnostic repose sur l'imagerie en coupe notamment l’échographie et le scanner qui permettent d'identifier le kyste, de préciser son siège, son type, ses dimensions et de mettre en évidence la communication avec la collection pariétale abdominale [5]. Le traitement est chirurgical consistant en une déconnection kysto-pariétale première avec évacuation de l'abcès intermédiaire puis du traitement du KH, de la cavité résiduelle. La prescription d'albendazole semble nécessaire pour éviter la récidive cutanée [4].

## Conclusion

La rupture du KH du foie dans la paroi abdominale est une complication exceptionnelle. L'imagerie joue un rôle important dans le diagnostic positif et la surveillance postopératoire. Les complications font la gravité de la maladie pouvant être évités la prévention de l'hydatidose et par le diagnostic et le traitement précoces du KH.
